# Value of Diffusion Weighted MRI with Quantitative ADC Map in Diagnosis of Malignant Thyroid Disease

**DOI:** 10.3390/diagnostics9040129

**Published:** 2019-09-25

**Authors:** Le Tuan Linh, Nguyen Ngoc Cuong, Tran Viet Hung, Nguyen Van Hieu, Bui Van Lenh, Nguyen Duy Hue, Van Huy Pham, Vu Thi Nga, Dinh-Toi Chu

**Affiliations:** 1 Radiology Division, Hanoi Medical University, Hanoi 100000, Vietnam; linhc.dhyhn2017@gmail.com (L.T.L.); buivanlenh@gmail.com (B.V.L.); d.huedhy@gmail.com (N.D.H.); 2 Radiology Department, Hanoi Medical University Hospital, Hanoi 100000, Vietnam; c.cdha@gmail.com (N.N.C.); h.tranbsb@gmail.com (T.V.H.); 3 Oncology Division, Hanoi Medical University, Hanoi 100000, Vietnam; hieu_nv.hmu@yahoo.com; 4 AI Lab, Faculty of Information Technology, Ton Duc Thang University, Ho Chi Minh City 700000, Vietnam; 5 Institute for Research and Development, Duy Tan University, Danang 550000, Vietnam; 6 Faculty of Biology, Hanoi National University of Education, Hanoi 100000, Vietnam; 7 School of Odonto Stomatology, Hanoi Medical University, Hanoi 100000, Vietnam

**Keywords:** quantitative ADC map, diagnosis, malignant thyroid disease, Vietnam

## Abstract

Thyroid nodule is a common disease in clinical practice. The diagnosis of malignant thyroid tumors determines the treatment strategy. Among a number of methods have claimed to help evaluating thyroid nodules, ultrasound is a usable one in spite of several disadvantages (dependent on the physician/technician, incomparable, etc.) and magnetic resonance imaging (MRI) accompanied by quantitative apparent diffusion coefficient (ADC) is a promising diagnostic tool. This study was designed to investigate the usefulness of ADC cut-off values and the protocol of thyroid MRI derived from quantitative diffusion weighted imaging (DWI) in differentiating benign and malignant thyroid nodules. The study was conducted on 93 patients with 128 thyroid nodules, diagnosed and underwent surgery at Hanoi Medical University Hospital. All the patients took thyroid MRI with different b levels (from 200 to 800). ADC value was calculated to each b level, and the statistical tests were conducted with the Statistical Package for Social Sciences (SPSS—Windows and Mac version 20) and STATA 12. The mean ADC with all the b ranging from 200 to 800 of malignant groups was significantly higher than the group of benign lesions (*p* from <0.001 to 0.01). We chose *b* = 500 as a standard b-value in the protocol of thyroid MRI. The ADC cut-off point for distinguishing malignant from benign thyroid lesions: 1.7 × 10^−3^ mm^2^/s with high accuracy (87.1%, 95% CI: 79.59–92.07%). The study revealed that quantitative diffusion weighted MRI with ADC measurement could potentially quantitatively differentiate between benign and malignant thyroid nodules.

## 1. Introduction

Thyroid nodules are a common entity and detected in 4–7% of the population by physical examination alone. However, the previous researches have shown 19–67% prevalence of thyroid nodules when examining the patients by modern imaging modality like ultrasound [1]. Thyroid nodules are clinically important as they may represent thyroid cancer in approximately 2.3% cases out of 100,000 Vietnamese women and 1.3% cases out of 100,000 Vietnamese men ranking 12th and 13th among the most frequent cancer forms in women and men respectively. Even though the incidence of thyroid nodules is fairly high, approximately 85% of the detected nodules are clinically insignificant benign lesions. The diagnostic imaging and nuclear medicine modalities are utilized to orient types of nodules and predict the invasiveness of thyroid cancer in situ, to the nodes and metastasis. Cytological and histopathological results help to establish a preoperative diagnosis and thyroid cancer classification [2,3]. In the past, patients with thyroid cancer used to have online neck examination by ultrasound and thyroid gland preoperative. Ultrasound is a noninvasive way to determine the suspicious lump, however it is not used for primary screening because a lot of factors. The result will be interpreted by a professional and the interpretation can vary depending on the evaluator. Quality of the machine could affect the result of examination [2,3]. In the recent literature, many authors have been deeply studying and analyzing the value of MRI in evaluating malignancy of thyroid [4,5,6]. With the advancement of high field magnetic resonance machines, especially the recent advanced pulse sequences, the oncology diagnosis of thyroid on MRI turns out to be a lot simpler. Diffusion-weighted imaging (DWI) is one of those advanced techniques. As a very quick examination which is non-invasive to make the image according to the Brownian motion of water molecule diffusion in the human body, DWI has been commonly utilized regular thyroid MRI. When taking the MRI exam, the targeted diffusion degree of DWI can be adjusted. b value is the index to indicate the diffusion degree of images, which could be calculated by the formula using some parameters of the magnetic machine (the gradient strength and the time between the two gradients). The diffusion characteristic of tissues only has light impact on the low b-value images. A higher b-value image comes along with noisier and much darker image (low signal to noise ratio). Most tissues drop their intensity from molecular motion, except for the restricted lesions. Normally, at least two images will be captured with different b values to acquire ADC value and to create ADC map image. ADC value strongly depends on the b-value image, which means ADC images are separately developed with different values of b. The ADC values reflect the properties of diffusion restriction of tissue. All the recent studies share one point of view, the authors highlight the advantages of the diffusion-weighted imaging (on the machines with strong magnetic field) which can measure the accurate value of ADC. It is necessary to establish the recommended cut-off point of ADC values to differentiate between malignant and benign lesions of thyroid. However, the authors still do not reach consensus about several contents, in particular about choosing the optimal b value for MRI exam of thyroid and the correspondent recommended cut-off ADC value to detect the malignant nodule [5].

On the other hand, currently in Vietnam, there are not any other former studies about the diffusion MRI in diagnosing malignancy of thyroid. Therefore, our purpose was to estimate the diagnostic value of quantitative DWI and calculate the cut-off ADC value to distinguish benign and malignant thyroid lesions using 1.5T MRI machines on patients with thyroid nodules in Vietnam.

## 2. Materials and Methods

### 2.1. Selection and Description of Participants

The study included 93 patients with 128 thyroid nodules, diagnosed and underwent surgery in Hanoi Medical University Hospital from 01/2013 to 12/2016. The selected patients have malignant suspected lesions of thyroid and are undergone preoperative MRI. The study excluded lesions with too small solid portion (<10 mm), because it was unable to place the regions of interest (ROI) to acquire ADC value.

All procedures performed in studies involving human participants were in accordance with the ethical standards of the institutional and/or national research committee and with the 1964 Helsinki declaration and its later amendments or comparable ethical standards. This study was approved by Vietnam Ministry of health with the identifycation code was 5297/QD-BYT issued on 25/12/2014. The study was also approved by the ethics committee of Vietnam Ministry of health. The patients were consulted and agreed to participate in the study. Informed consent was obtained from the patient included in the study.

### 2.2. Technical Information and Statistics

**Figure 1:**
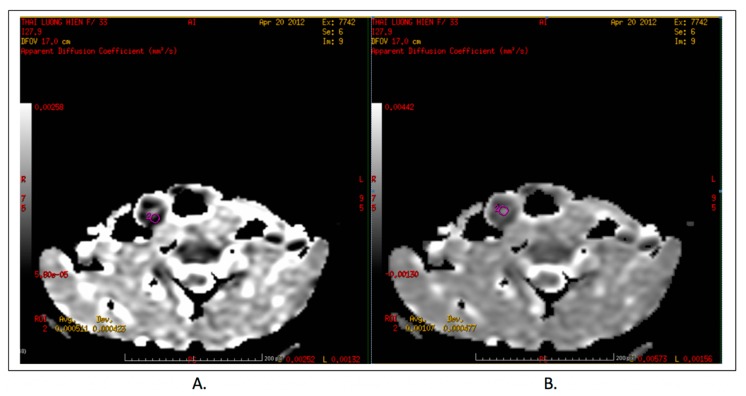
Demonstration on measuring ADC values accurately: (**A**) Right method. (**B**) Wrong method.

In this study, Signa Hdxt1.5T (GE Healthcare, Chicago, IL, USA) was used for examination on MRI with 8 N-VARRAX A coils, using 25–30 cm field of view and 6mm slice thickness, on axial plane. The ADC value was measured on different b-value (200, 300, 400, 500, 600, 700, 800), the ROI were placed in the solid part of the lesions (not at the colloid cyst, nor the macro calcification) with the lowest ADC value. Figure 1 shows how to measure ADC correctly.

All the ADC values of two groups were checked with histopathology results (proved benign and proved malignant nodules). The values were demonstrated as mean and standard deviation. The area under the curve receiver operating characteristic (ROC) was also calculated. From ROC curve analysis on the b value, an optimal cut-off value of ADC to diagnose malignant lesions of thyroid was determined by using Youden’s index. Based on this data-driven cut-off value, sensitivity, specificity, positive predictive value (PPV), negative predictive value (NPV), and accuracy were assessed and supplemented with 95% confidence intervals (95% CI). The statistical tests were calculated on personal computers utilizing the Statistical Package for Social Sciences (SPSS—Windows and Mac version 20) and STATA 12.

## 3. Results

Our study was conducted on 93 patients with a total of 128 thyroid nodules, 49 malignant lesions (38.3%) and 79 benign lesions (61.7%). There are 2 patients with 2 malignant thyroid tumors on each. Mean diameter of malignant lesions was 21.61 ± 13.22 mm, of benign lesions were 24.61 ± 12.22 mm. There is no significant difference in size between the 2 groups (*p* = 0.36).

The higher b value, the lower signal to noise ratio, and some thyroid nodules can be missed on the images with high b-value (128 nodules at b of 200–500, 123 nodules at b of 600, 116 nodules at b of 700 and 100 nodules at b of 800). All the mean value of ADC with correspond b value for each group of patients are expressed in the Table 1. There was significant difference between mean ADC value of benign and malignant nodule group on all images from b200 to b800 (*p* < 0.001 to 0.01, see the Table 1).

As we can see in Table 2, with each b-value, we created the curve ROC and calculated the area under the curve; therefore, the discriminatory power of the ADC value to predict the benignity or malignancy of thyroid tumors was determined. Area under the curve ROC are all close to 1 (0.9–0.94), that means the test with any b-value are highly practical.

**Figure 2:**
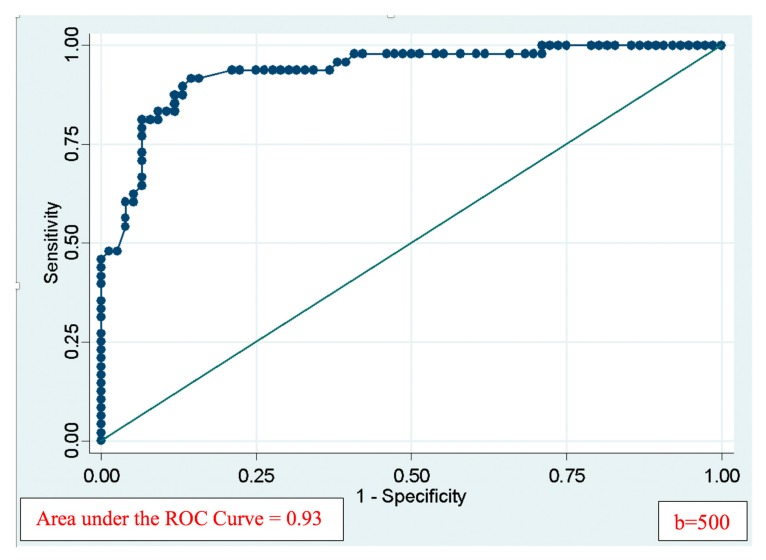
The ROC curve with b = 500, the area under ROC curve = 0.93.

ROC curve with b = 500 is presented in Figure 2, with the area under the curve is 0.93. Similar figures were also created with other b values, but in this article only the value *b* = 500 is included into the chart (others is available in a supplementary file to this article). This optimal b value is considered to be optimal choice, which will be explained in detail in Discussion.

Finally, all the statistical measures of the performance of the test with various b-value are shown on Table 3. The sensitivity (78.57% to 84.38%) and the PPV (79.17% to 87.1%) are limited. As a consequence, the specificity, NPV are quite high when differentiating benign and malignant tumors of the thyroid gland by using ADC value.

## 4. Discussion

**Figure 3:**
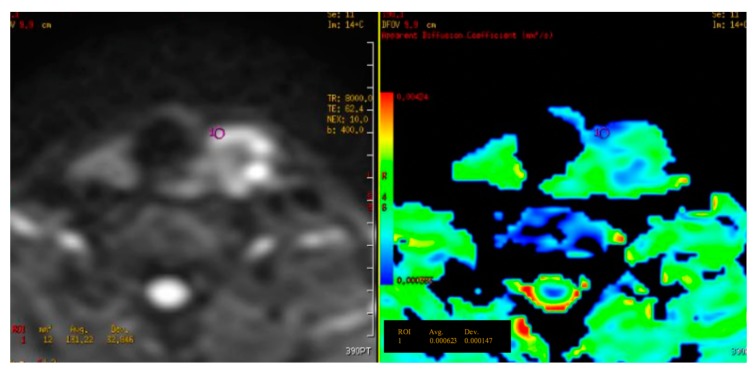
Mean ADC value of a malignant thyroid nodule with b = 800 is 0.62 × 10^−3^ mm^2^/s. Histopathology result: Papillary carcinoma.

**Figure 4:**
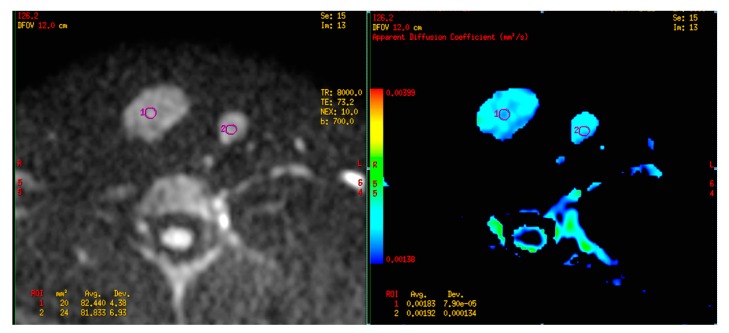
Right and left lobe thyroid nodules, with b = 700, ADC: 1.83 × 10^−3^ mm^2^/s and 1.92 × 10^−3^ mm^2^/s. Histopathology result: benign nodules.

In our study, all the thyroid lesions are diffusion restricted (high signal on DWI, low value ADC). Therefore, unlike tumors in other organs, the benign and malignant thyroid nodules have similar signal on DWI. It is impossible to just compare the signal of thyroid nodules on diffusion sequences or ADC map to diagnose malignant tumors. The diagnosis can only be made with quantitative ADC. Figure 3 and Figure 4 show us the two typical malignant and benign lesions with the very different ADC value. In this study, with all the b range from 200 to 800, the average ADC value of malignant nodules of thyroid were all significantly higher than the benign group (*p* < 0.001 to *p* = 0.01). These statistically significant findings support the role ADC value as an important index to figure out which is a malignant nodule on a thyroid MRI. This result is similar to some other authors, for example, Bozgeyik Z. (2009) measured 93 thyroid nodules and indicated a statistically significant difference between the benign and malignant group in term of ADC value, at b100, b200 and b300 [7]. Similarly, Noda Y. (2015) acquired the images of thyroid at b0 and b1000, obtaining the average ADC value of benign lesions is (1.88 ± 0.5) × 10^−3^ mm^2^/s; of malignant tumors is (0.89 ± 0.1) × 10^−3^ mm^2^/s (significant difference with *p* < 0.05) [6].

The ADC value is variable and depends on diffusion factor b. As the b value rises, the tests become more specific. However, when b factor is elevating, the noise is increasing too, and the number of observed nodules is decreased. With factor b from 200 to 500, all thyroid nodules are visible on DWI and can be measured ADC value. With b600, b700 and b800, the number of missing nodules was 5/128, 12/128 and 28/128 respectively. In addition, at high b, there is an increase in signal to noise ratio, placement of ROI (region of interest) on the lesions is also more likely to cause errors, especially with lesions below 10mm. In a meta-analysis by Chen L. (2016) with 15 different studies, each author chose only one b-value (Include b300, b500 and b1000). However, Chen has not analyzed yet to find out the best b value for thyroid MRI. The suitable b value must have both acceptable diffusion characteristic and fairly low noise (to not miss the lesions) [5]. Recently, with the advanced MRI technique, the trend of using high value b is applied by many authors to distinguish the benign/malignant nature of the injury, with b800–1000; the average accurate diagnosis rate is 96% [5]. However, in Vietnam, most of MRI machine are 1.5 T, it is necessary to balance the benefit of the augmentation of b value with the capable of the device. We suggest with 1.5T MRI machine, it is necessary to find out the most valuable b value to reduce the time of MRI exam (which is quite uncomfortable).

In our research, the area under the curve ROC of the test using ADC value to diagnose malignant thyroid nodules was very close to 1 (90–94%) with all different b values (from 200 to 800). It showed that quantitative ADC value may be helpful in this differentiation. As we can see at the Table 2, when the value of b is increasing the area under the curve ROC tends to be higher. With b range from 200 to 500, the area under the curve is all acceptable for a good test (>0.9). Therefore, it is unnecessary to perform MRI with various b values (which takes a lot of time). Besides, we also recommend not taking MRI with too high b value because the lesions will become difficult to observe. The optimal b value is the highest b value obtained without missing the lesions. The b500 value was chosen as the only applicant b value on thyroid MRI for 2 reasons. First, according to the meta-analysis of Chen L. (2016) the accurate rate of diagnosis are elevating as the b value is increasing [5]. Chen also concluded in the study on the assessment of the value of diagnosis of neck lymph node metastasis, only the pulse sequences with b value of 500 or more are significant for detecting lymph nodes. For these reasons, the diffusion sequence at b500 can be used for two purposes: to make a differential diagnosis benign and malignant thyroid lesions, and to detect metastasis lymph glands. With b500 diffusion imaging, the average ADC value of benign group was (1.22 ± 0.58) × 10^−3^ mm^2^/s; malignant group (2.05 ± 0.44) × 10^−3^ mm^2^/s (*p* < 0.001). About quantitative ADC test for malignant lesions, the cut-off point of value ADC is 1.7 × 10^−3^ mm^2^/s; with the area under the curve ROC was 0.93; the sensitivity 81.25%; the specificity 90.79%, PPV 84.78%, NPV 88.46%, and the accuracy 87.1%. Comparing with some other authors, such as Razek A. (2008) [8], studied 67 patients who concluded that the average ADC of thyroid cancer was (0.73 ± 0.19) × 10^−3^ mm^2^/s, the difference was statistical significant compared to the mean value of benign tumor group ((1.8 ± 0.27) × 10^−3^ mm^2^/s), *p* < 0.0001. The cut-off point of ADC was 0.98 × 10^−3^ mm^2^/s, the degree sensitivity, specificity, accuracy was 97.5%, 91.7% and 98.9%; respectively [8]. Srinivasan A. (2008) [9] investigated on 33 patients taking MRI 3T with neck tumors, including thyroid tumors and found out the mean ADC value of the malignant tumors was (1.071 ± 0.293) × 10^−3^ mm^2^/s, lower compare to that index of benign lesions ((1.505 ± 0.487) × 10^−3^ mm^2^/s), with significant difference *p* < 0.004, cut-off point was 1.3 × 10^−3^ mm^2^/s [9]. Nakahira M. (2012) [10] studied 38 patients with 42 thyroid nodules that concluded that there was significant difference in ADC values between the benign and malignant groups. With the cut-off point of ADC is 1.60 × 10^−3^ mm^2^/s, the sensitivity, the specificity and the accuracy is 94.73%; 82.60%; 88.09%, respectively [10]. Chen L. (2016), on his recent meta-analysis study, reviewed 765 thyroid nodules with measured ADC (b100 to b1000), apply on both MRI 1.5T and 3T. The authors found that the average sensitivity and specificity of diffuse MRI when differentiating benign and malignant thyroid nodules were 90% (from 79% to 97%) and 95% (from 79% to 100%) [5]. In summary, although mean ADC value of benign/malignant nodules group and the cut-off points are different (because of the variety of MRI machine and b value), all the authors made a conclusion that quantitative ADC map is a valuable test to determine malignant thyroid nodules.

However, our prospective study has some limitations. First, our cohort of patients was small and therefore ADC values obtained for differential diagnosis of malignant and benign lesions of thyroid need to be confirmed in other trials with larger number of patients. Secondly, the measurement is not quite accurate with such small lesions because of the low spatial resolution of the image (which is the nature of the DWI technique with 1.5T MRI machine). In the near future, with technical progression, these disadvantages can be overcome. Finally, the most optimized chosen b-value in our study with such MRI machine should be verified in further studies.

## 5. Conclusions

MRI with ADC measurement value in DWI is very useful in diagnoses the malignancy of thyroid nodules. With the higher b-value, ADC is more valuable to distinguish benign and malignant lesions of thyroid. However, for not missing the lesions under 10mm, it is recommended to use DWI with b500, ADC cut-off is 1.7 × 10^−3^ mm^2^/s (Accuracy 87.1%, Specificity 90.79%).

## Figures and Tables

**Table 1:** Compare mean ADC value of two groups: malignant and benign lesions with different b-value on Diffusion Weighted MRI.

*b* ADC	200 (*n*= 128)	300 (*n* = 128)	400 (*n* = 128)	500 (*n* = 128)	600 (*n* = 123)	700 (*n* = 116)	800 (*n* = 100)
Benign (×10^−3^ mm^2^/s)	1.38 ± 0.36	1.37 ± 0.32	1.3 ± 0.35	1.22 ± 0.38	1.20 ± 0.34	1.18 ± 0.43	1.08 ± 0.34
Malignant (×10^−3^ mm^2^/s)	2.27 ± 0.45	2.21 ± 0.47	2.13 ± 0.47	2.05 ± 0.44	1.98 ± 0.45	1.92 ± 0.43	1.98 ± 0.4
*p*	<0.001	<0.001	<0.001	<0.001	0.01	<0.001	<0.001

**Table 2:** Area under the curve ROC with each b-value in using quantitative ADC for distinguishing benign and malignant thyroid tumors.

*b*-Value	200 (*n* = 128)	300 (*n* = 128)	400 (*n* = 128)	500 (*n* = 128)	600 (*n* = 123)	700 (*n* = 116)	800 (*n* = 100)
Area	0.92	0.93	0.92	0.93	0.92	0.9	0.94
95% CI	0.88–0.99	0.87–0.98	0.86–0.98	0.88–0.99	0.86–0.98	0.82–0.98	0.88–0.99

**Table 3:** Cut-off point of ADC with each b factor and value of the diagnosis.

*b*-Value	200 (*n* = 128)	300 (*n* = 128)	400 (*n* = 128)	500 (*n* = 128)	600 (*n* = 123)	700 (*n* = 116)	800 (*n* = 100)
Cut-off ADC (×10^−3^ mm^2^/s)	1.86	1.88	1.75	1.70	1.68	1.62	1.51
Sn	81.08%	81.25%	79.17%	81.25%	84.78%	78.57%	84.38%
95% CI of Sn	66.74–90.85%	67.98–91.24%	65.66–89.76%	67.98–91.24%	72.24–93.93%	64.34–89.30%	68.75–93.98%
Sp	90.48%	90.91%	87.01%	90.79%	91.55%	88.52%	92.59%
95% CI of Sp	81.46–95.64%	82.59–96.36%	77.95–93.76%	81.02–95.53%	83.40–97.01%	78.43–94.86%	84.30–98.21%
PPV	83.33%	84.78%	79.17%	84.78%	86.67%	82.50%	87.10%
95% CI of PPV	70.80–90.30%	73.56–92.15%	68.24–87.62%	71.89–90.72%	75.87–93.69%	70.33–90.02%	75.43–95.42%
NPV	89.06%	88.61%	87.01%	88.46%	90.28%	85.71%	90.91%
95% CI of NPV	81.78–93.61%	81.54–93.55%	79.77–92.35%	81.31–93.46%	83.21–95.15%	77.76–91.41%	82.22–95.28%
Acc	87%	87.2%	84%	87.1%	89%	84.5%	90%
95% CI of Acc	79.59–92.07%	80.50–92.68%	76.91–90.19%	79.59–92.07%	82.60–94.25%	76.59–90.54%	82.38–95.10%

## Abbreviations

ADC	Apparent diffusion coefficient
MRI	Magnetic resonance imaging
DWI	Diffusion weighted imaging
ROC	Receiver operating characteristic
ROI	Regions of interest
Sn	Sensitivity
Sp	Specificity
PPV	Positive predictive value
NPV	Negative predictive value
Acc	Accuracy
